# A Survival Scoring System for Non-Small Cell Lung Cancer Patients with *De Novo* Bone Metastases

**DOI:** 10.1371/journal.pone.0167923

**Published:** 2016-12-08

**Authors:** Yu-Mu Chen, Ying-Tang Fang, Chien-Hao Lai, Kun-Ming Rau, Cheng-Hua Huang, Huang-Chih Chang, Tung-Ying Chao, Chia-Cheng Tseng, Wen-Feng Fang, Chin-Chou Wang, Yung-Che Chen, Yu-Hsiu Chung, Yi-Hsi Wang, Mao-Chang Su, Shih-Feng Liu, Kuo-Tung Huang, Hung-Chen Chen, Ya-Chun Chang, Yu-Ping Chang, Meng-Chih Lin

**Affiliations:** 1 Division of Pulmonary and Critical Care Medicine, Department of Internal Medicine, Chang Gung Memorial Hospital-Kaohsiung Medical Center, Chang Gung University College of Medicine, Kaohsiung, Taiwan; 2 Division of Hematology-Oncology, Department of Internal Medicine, Kaohsiung Chang Gung Memorial Hospital and Chang Gung University College of Medicine, Kaohsiung, Taiwan; 3 Department of Respiratory Care, Chang Gung University of Science and Technology, Chiayi Campus, Chiayi, Taiwan; 4 Graduate Institute of Clinical Medical Sciences, Chang Gung University College of Medicine, Kaohsiung, Taiwan; Western General Hospital, UNITED KINGDOM

## Abstract

In the pre-tyrosine kinase inhibitors (TKIs) era, non-small cell lung cancer (NSCLC) patients with *de novo* bone metastases had a worse prognosis than those without. However, whether epidermal growth factor receptor (EGFR)-TKIs affect the outcomes of *EGFR* mutant NSCLC patients with *de novo* bone metastases has not been well studied thus far. We retrospectively studied the effect of *EGFR* mutation status and first-line EGFR-TKIs on patient outcomes and created a survival scoring system for NSCLC patients with *de novo* bone metastases. This retrospective study evaluated 1510 NSCLC patients diagnosed between November 2010 and March 2014. Among these patients, 234 patients had *de novo* bone metastases. We found that 121 of these 234 patients (51.7%) had positive *EGFR* mutation tests, and a positive *EGFR* mutation test significantly affected overall survival (OS) (*EGFR* mutant: 15.2 months, *EGFR* wild type: 6.5 months; p < 0.001). Other prognostic factors significant in the multivariable analysis for NSCLC with *de novo* bone metastases included Eastern Cooperative Oncology Group performance status (PS) (OS; PS 0–2: 11.2 months, PS 3–4: 4.9 months; p = 0.002), presence of extraosseous metastases (OS; with extraosseous metastases: 8.8 months, without extraosseous metastases: 14.0 months; p = 0.008), blood lymphocyte-to-monocyte ratio (LMR) (OS; LMR > 3.1: 17.1months, LMR ≤ 3.1: 6.9months; p < 0.001). A positive *EGFR* mutation status reversed the poor outcomes of NSCLC patients with *de novo* bone metastases. A simple and useful survival scoring system including the above clinical parameters was thus created for NSCLC patients with *de novo* bone metastases.

## Introduction

Lung cancer is the leading cause of cancer death worldwide and in Taiwan.[1, 2] Today, a further understanding of the molecular mechanisms underlying non-small cell lung cancer (NSCLC) has resulted in the development of epidermal growth factor receptor (EGFR)-tyrosine kinase inhibitors (TKIs). Previous studies showed that EGFR-TKIs improved quality of life, progression-free survival (PFS), and even overall survival (OS) in advanced NSCLC patients harboring *EGFR* mutations.[3–5]

However, as the most common cause of cancer-related pain, bone metastases significantly reduce quality of life.[6] Bone metastases occur in 24.0–39.8% of NSCLC patients, most commonly involving the weight bearing skeleton and proximal long bones.[7, 8] Bone metastases also lead to skeletal-related events (SREs), which can negatively affect quality of life and survival duration.[9] The median survival duration for patients with bone metastases is often less than 1 year.[10–12]

The lung has been demonstrated to be the primary cancer site in more than 50% of cases of unknown primary cancer with bone metastases at autopsy.[13] Among patients with metastatic spinal cord compression (MSCC) secondary to cancer with bone metastases, lung cancer has a worse prognosis than MSCC related to other solid tumors.[14–16] Although several bone-directed targeted therapies have been introduced, these patients’ prognosis is still poor.[10]

The aim of this study was to determine if patients positive for *EGFR* mutations who were taking first-line EGFR-TKIs experienced a reversal of the poor outcomes of NSCLC patients with *de novo* bone metastases. We also investigated other clinical factors affecting outcomes in these patients, and created a simple and useful survival scoring system.

## Materials and Methods

### Patient and clinical characteristics

This retrospective study evaluated patients with NSCLC who were diagnosed between November 2010 and March 2014 at Kaohsiung Chang Gung Memorial Hospital in Taiwan. All patients were subsequently followed-up until November 2015. The inclusion criteria were age >18 years, histologically or cytologically confirmed advanced-stage NSCLC with *de novo* bone metastases, and having undergone an *EGFR* mutation test.

We used Tc99−bone scans with chest radiography and computed tomography for initial evaluation of the presence of bone metastases. If the results were conflicting between the clinical physicians and nuclear medicine physicians, we held a joint meeting for consensus. If bone was the only possible distant metastasis site, and it was feasible to perform curative surgery after excluding bone metastasis, a positron emission tomography scan was performed for further evaluation. Sometimes, bone metastases were the initial presentation of advanced NSCLC and were diagnosed via bone biopsy samples.

Patients were excluded if they had previously received any targeted therapy, chemotherapy, or immunotherapy. Patients were excluded if they refused to take EGFR-TKIs when they were positive for *EGFR* mutations. This study’s design was approved by the Institutional Review Board of Kaohsiung Chang Gung Memorial Hospital, and the requirement for informed consent was waived due to the retrospective design.

Baseline assessments were performed within 4 weeks of treatment initiation, including clinical characteristics and findings from chest radiography, chest computed tomography, bone scan, and brain magnetic resonance imaging. The clinical characteristics included age, body mass index (BMI), sex, smoking status, blood lymphocyte-to-monocyte ratio, Eastern Cooperative Oncology Group performance status (PS), diabetes mellitus, *EGFR* mutations, tumor histology, and sites and symptoms of distant metastases.

### *EGFR* mutation testing

Tumor specimens were obtained from biopsy samples that were obtained via bronchoscopy, computed tomography-guided biopsy, or surgical procedures. Tumor specimens from pleural effusion cytology were also considered acceptable. The genetic analyses were performed using Scorpion primers and genomic DNA that was extracted from the paraffin-embedded tissues (QIAGEN EGFR RGQ PCR Kit), which were subjected to amplification refractory mutation system-polymerase chain reaction.[17]

### Evaluating response to EGFR-TKI treatment

To evaluate the tumor response, patients underwent chest radiography every 2–4 weeks and chest computed tomography every 2–3 months. Disease status was determined by the attending clinician according to Response Evaluation Criteria in Solid Tumors guidelines (version 1.1).[18] Overall survival (OS) was defined as the period from the first day of EGFR-TKI treatment until death, loss to follow-up, or the last follow-up.

### Statistical analyses

Statistical analyses were performed using MedCalc software (version 14.10.2). The OS analyses were performed using the Kaplan-Meier method and the log-rank test. A Cox proportional hazards regression model was used to evaluate independent factors that affected the survival outcomes. Receiver operating characteristic (ROC) curves were drawn and Youden's index was used to determine the best cut-off value for LMR, the area under the curve, sensitivity and specificity of the survival scoring system. A p value of <0.05 was considered statistically significant.

## Results

### Patient characteristics

**Fig 1:**
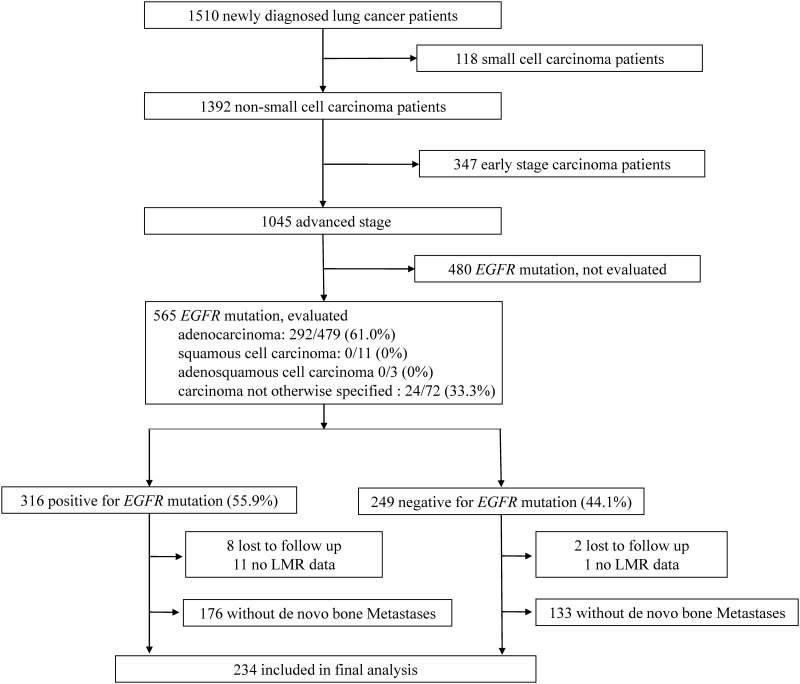
The inclusion and screening for this study. Among 1,510 patients who were diagnosed with non-small-cell lung cancer between November 2010 and March 2014, 234 patients with *de novo* bone metastases were included in the final analysis.

Among 1510 patients who were diagnosed with lung cancer between November 2010 and March 2014, we identified 234 NSCLC patients with *de novo* bone metastases (Fig 1). Among these patients, 51.7% (121/234) patients had positive *EGFR* mutation tests. The mean patient age was 61.9 ± 13.0 years and 43.6% (102/234) of the patients were men. At the last follow-up, 13.2% (31/234) of the patients were alive. The median OS was 10.5 ± 1.0 months, the longest follow-up was 54.8 months. The best cut-off point of LMR determined by ROC curve and Youden’s Index was 3.1. Patients were divided into high or low LMR based on above cut-off value. The first-line therapy for study patients were listed in S1 Table.

### Survival analysis

#### Survival analysis according to distant metastatic sites

**Fig 2:**
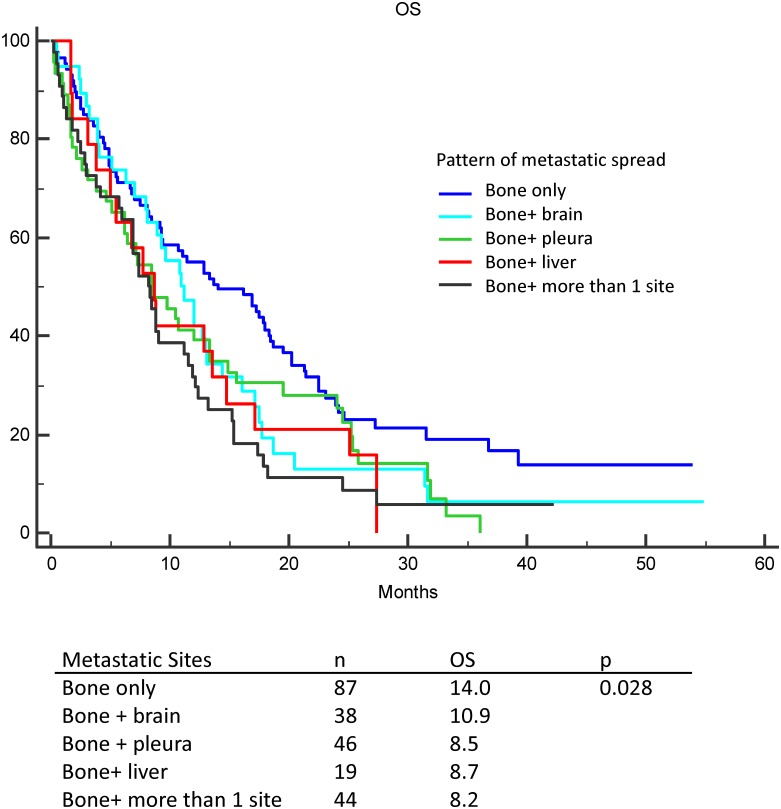
Kaplan-Meier curve for overall survival according to patterns of metastatic spread. Patients with bone metastases only had longer OS duration than those concomitant with other metastatic sites. (p = 0.028)

Besides 87 patients had bone metastases only, 147 patients had other concomitant distant metastases at diagnosis. (Fig 2) Of the 147 patients, 38 patients had concomitant brain metastases, 46 patients had concomitant pleura metastases, 19 patients had concomitant brain metastases, and 44 patients had more than 2 concomitant distant metastases. Patients with bone metastases only had longer OS duration than those concomitant with other metastatic sites.

#### Survival analysis for patients with *de novo* bone metastases

In the univariable analysis, prolonged OS was significantly associated with female sex (p = 0.001), absence of diabetes mellitus (p = 0.007), never smoker (p = 0.007), a PS of ≤2 (p < 0.001), a positive *EGFR* mutation test result (p < 0.001), no extraosseous metastasis (p = 0.003), and LMR > 3.1 (p < 0.001) (Table 1). Age, body mass index, and tumor histology were not significantly associated with OS. In the multivariable analysis, prolonged OS was independently associated with a PS of ≤2 (p = 0.002), positive *EGFR* mutation test results (p = 0.004), no extraosseous metastasis (p = 0.008), and LMR > 3.1 (p < 0.001) (Table 1). These Four prognostic factors were included in the scoring system (Table 2).

**Table 1:** Impact of baseline clinical parameters on overall survival of NSCLC patients with *de novo* bone metastases.

	Univariate analysis			Multivariate analysis		
	n	OS (months)	P value	Hazard ratio	P value	95% CI
Age, years			0.135		0.102	
>60	127	8.8		1.29		0.951–1.753
≤60	107	12.1		1.00		
BMI			0.173		0.795	
>22	122	12.4		1.00		0.764–1.421
≤22	112	9.0		1.04		
Sex			0.001		0.337	
Male	102	8.4		1.24		0.802–1.902
Female	132	12.0		1.00		
DM			0.007		0.120	
YES	22	5.5		1.49		0.902–2.455
NO	212	10.9		1.00		
Smoking history			0.007		0.449	
Never	155	12.0		1.00		0.753–1.897
Former / current	79	8.2		1.12		
Performance status			<0.001		0.002	
ECOG 0–2	203	11.2		1.00		1.313–3.352
ECOG 3–4	31	4.9		2.10		
EGFR Mutation			<0.001		0.004	
Yes	121	15.2		1.00		1.161–2.212
No	113	6.5		1.60		
Tumor type			0.248		0.923	
Adenocarcinoma	194	10.8		1.00		0.685–1.518
Non-adenocarcinoma	40	9.3		1.02		
Extraosseous metastasis			0.003		0.008	
Yes	147	8.8		1.59		1.131–2.245
No	87	14.0		1.00		
LMR			<0.001		<0.001	
>3.1	112	17.1		1.00		1.404–2.679
≤3.1	122	6.9		1.94		

BMI, body mass index; DM, diabetes mellitus; ECOG, Eastern Cooperative Oncology Group; EGFR, epidermal growth factor receptor; LMR, lymphocyte-to-monocyte ratio; NSCLC, non-small cell lung cancer; OS, overall survival

**Table 2:** Scoring system for NSCLC with bone metastasis.

	Score
Performance status	
ECOG 0–2	0
ECOG 3–4	1
EGFR Mutation	
Yes	0
No	1
Extraosseous metastasis	
Yes	1
No	0
LMR	
>3.1	0
≤3.1	1

ECOG, Eastern Cooperative Oncology Group; EGFR, epidermal growth factor receptor; LMR, lymphocyte-to-monocyte ratio; NSCLC, non-small cell lung cancer

### Survival scoring system

**Fig 3:**
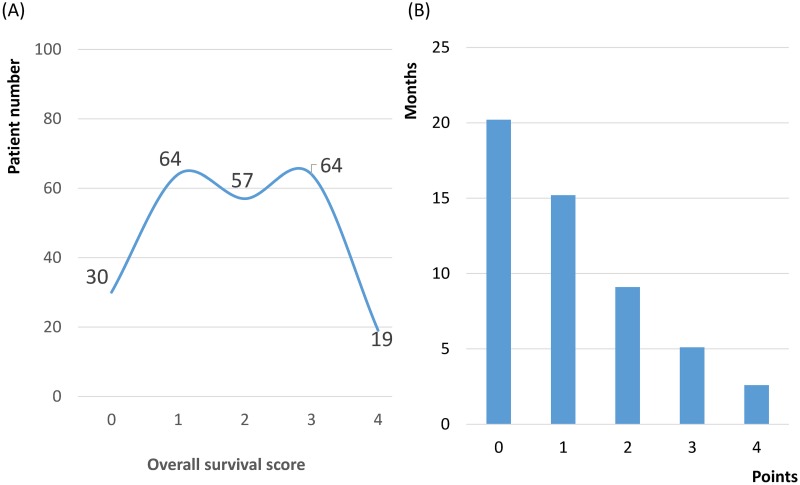
(A) Distribution of survival scores over the 234 patients; (B) Median overall survival related to the corresponding scores.

**Fig 4:**
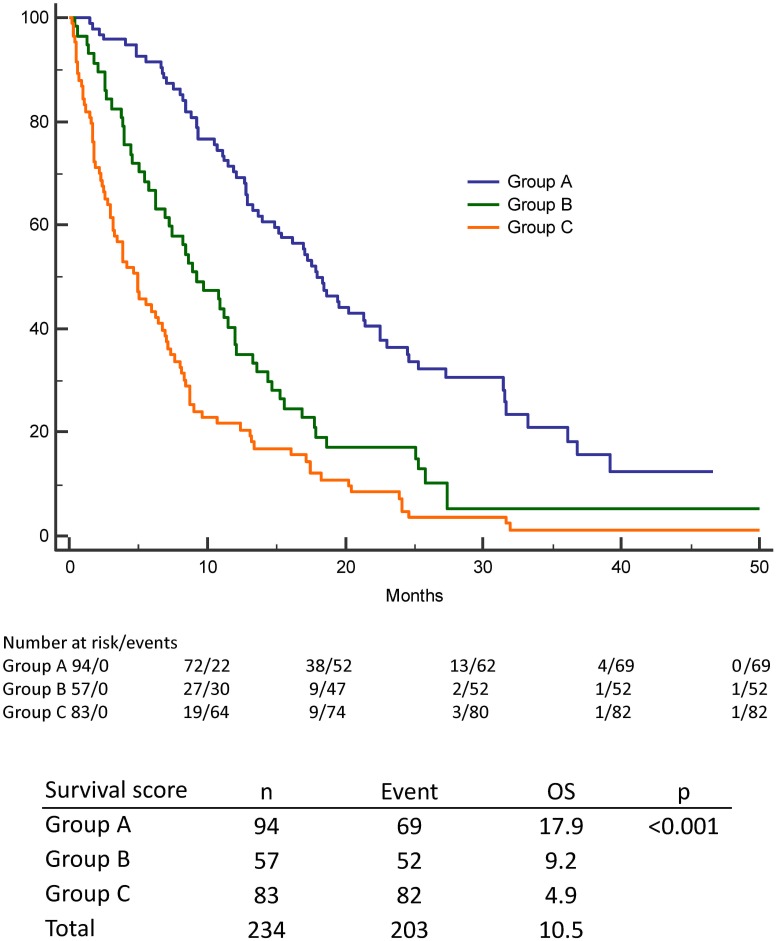
Kaplan-Meier curve for overall survival of the three prognostic groups A, B, and C. OS among NSCLC patients with *de novo* metastases according to three prognostic groups A, B, and C.

The scoring system ranged from 0 to 4. The distribution of survival scores over the 234 patients is shown in Fig 3A The median OS duration was 20.2 months for patients with 0 points, 15.2 months for patients with 1 point, 9.2 months for patients with 2 points, 5.1 months for patients with 3 points, and 2.6 months for patients with 4 points (Fig 3B). Three prognostic groups were identified according to the total score: 0–1 points (group A), 2 points (group B), and 3–4 points (group C). The median OS was 17.9 month in group A, 9.2 month in group B, and 4.9 month in group C (p < 0.001) (Fig 4).

## Discussion

Previous studies revealed patients with adenocarcinoma were more likely to have bone metastases than those with small cell lung cancer, and those with squamous cell carcinoma were less likely to have bone metastases.[19, 20] Fortunately, adenocarcinoma was more likely to have *EGFR* mutations than other histologic types.[21] In a previous study, among NSCLC patients, more than 35.3% of patients with bone metastases exhibited bone involvement at the time the lung cancer was diagnosed.[10] Our study revealed 43.8% (243/555) patients had *de novo* bone metastases, and we included only patients with *de novo* bone metastases to decrease confounding factors. In patients receiving platinum-based chemotherapy, the outcomes of NSCLC patients with bone metastases is poor, with a median survival of less than a year.[22–24] Our study revealed EGFR-TKIs had prolonged the median survival duration from 6.5 months to 15.4 months.

In pre-TKIs era, young advanced NSCLC patients had better prognosis than older patients.[25] However, in TKIs era, previous study showed that young lung cancer patients with positive EGFR mutant test had only 3.3 months PFS when receiving first-line EGFR-TKIs. [26] Regardless of EGFR mutation status, our study revealed younger or older than 60 years old NSCLC patients with de novo bone metastases had equivalent OS length. Higher BMI were found prognostic factor in advanced NSCLC patients receiving chemotherapies.[27] Although our study revealed *de novo* bone metastases patients with BMI > 22 had a 3.4 months OS prolongation than those with BMI ≤ 22, the result did not meet statistical significance. Female, non-smoker and adenocarcinoma histology were predictor of having positive EGFR mutation. [28–31] However, our study revealed they were not prognostic factors in NSCLC patients with *de novo* bone metastases.

Previous studies revealed lung cancer patients with good PS had better outcomes when receiving chemotherapies or first-line EGFR-TKIs.[26, 32, 33] Consistent with previous studies, our study revealed patients with bone metastases had better survival if they had a good PS.[22] We assume patients with poor PS are not appropriate for chemotherapies, radiotherapies, or palliative surgery; experience more myelosuppression; are more likely to succumb to non-cancer death; and consequently have dismal outcomes.[34]

Previous studies have shown that the presence and number of distant metastases are prognostic factors for NSCLC patients treated with chemotherapy or EGFR-TKIs.[26, 35–37] Our study revealed that patients with bone metastases only had better outcomes than those who had distant metastasis at sites other than bone.

Previous revealed LMR was prognostic factor in early-stage malignancies including lung cancer[38], and nasopharyngeal carcinoma [39]; in advanced-stage malignancies including diffuse large B-cell lymphoma [40, 41], lung cancer patients receiving chemotherapies[42], and breast cancer patients receiving neo-adjuvant chemotherapies.[43] Our study revealed that patients with *de novo* bone metastases had better prognosis if they had higher baseline blood LMR ratio. To the best of our knowledge, this is the first study demonstrating that LMR is a prognostic factor in NSCLC patients with *de novo* bone metastases.

The present study had several limitations. First, we did not have access to the use of bone-directed targeted therapies. Whether these agents have a synergic effect with EGFR-TKIs cannot be explored. However, most patients started receiving bone-directed agents when they had first time SREs in our institute and a first SRE often heralds subsequent SREs. Thus, we decided not to evaluate the impact of bone-directed agents on survival outcome to prevent bias. Second, our study used a retrospective design with a small patient population, and prospective studies are needed to validate our findings.

## Conclusion

Positive *EGFR* mutation status reversed the poor outcomes of NSCLC patients with *de novo* bone metastases. A simple and useful survival scoring system was thus created for NSCLC patients with *de novo* bone metastases.

## Supporting Information

S1 Table First-line therapy of study patients. (DOC)

S1 Data Raw data file. (SAV)
