# Supplementary material for: A Survival Scoring System for Non-Small Cell Lung Cancer Patients with De Novo Bone Metastases
Source: PLoS One. 2016 Dec 8;11(12):e0167923. doi: 10.1371/journal.pone.0167923 (PMC5145216; doi:10.1371/journal.pone.0167923)
Supplement: S1 Table — (DOC) [file pone.0167923.s001.doc]

**S1 Table. First-line therapy of study patients.**

| EGFR mutant patients | N=121 |
| --- | --- |
| Gefitinib | 107 (88.4%) |
| Erlotinib | 14 (11.6%) |
| EGFR wild type patients | N=113 |
| Platinum* + pemetrexed | 37 (32.7%) |
| platinum + gemcitabine  platinum + Docetaxel  platinum + paclitaxel  platinum + vinorelbine  Pemetrexed  Gemcitabine  Docetaxel  Vinorelbine  Palliative care | 6 (5.3%)  25 (22.1%)  2 (1.8%)  3 (2.7%)  6 (5.3%)  5 (4.4%)  8 (7.0%)  6 (5.3%)  15 (13.3%) |

*Carboplatin was prescribed if CCR<60; otherwise, we use cisplatin as first-line therapy
